# A Mobile Wireless Sensor Coverage Optimization Method for Bridge Monitoring

**DOI:** 10.3390/s25092772

**Published:** 2025-04-27

**Authors:** Cong Mu, Jiguang Yang, Jiuyuan Huo

**Affiliations:** 1 School of Electronic and Information Engineering, Lanzhou Jiaotong University, Lanzhou 730070, China; mucong@lzjtu.edu.cn (C.M.); 0619664@stu.lzjtu.edu.cn (J.Y.); 2 National Cryosphere Desert Data Center, Northwest Institute of Eco-Environment and Resources, CAS, Lanzhou 730000, China

**Keywords:** heterogeneous mobile wireless sensor, hiking optimization algorithm, coverage optimization, bridge monitoring

## Abstract

Aiming at the problem of resource allocation optimization of heterogeneous mobile wireless sensor (HMWS) networks in bridge structural health monitoring, this study proposes an enhanced coverage strategy for heterogeneous sensors based on an improved Hiking Optimization Algorithm (HOA). This paper integrates the Good Point Set theory with a heterogeneous degree-of-freedom t-distribution perturbation mechanism to improve the basic HOA, developing the GPTHOA with global optimization characteristics. Based on this, a virtual force-guided HMWS coverage enhancement strategy (the VF-GPTHOA) is proposed. After determining the optimal deployment scheme, the GPTHOA is further employed to optimize node movement trajectories, minimizing the movement distance. Simulation results show that when deploying 60 heterogeneous sensors of three types in a 40 m × 200 m bridge model (BM), the coverage rate (CR) of the VF-GPTHOA reaches 97.07%, which represents improvements of 13.81%, 4.18%, 15.81%, 2.52%, and 12.44% over DE, MA, GWO, SSA, and HOA, respectively. In dynamic node scale scenarios, the VF-GPTHOA maintains optimal coverage performance, demonstrating its robustness and applicability in engineering practice.

## 1. Introduction

As critical components of modern transportation systems, bridges constitute essential infrastructure that directly influences national economic development and public safety. Throughout their designed service lifespan, these civil engineering structures must maintain structural integrity and serviceability while withstanding cumulative traffic loads and extreme environmental challenges, including seismic events, hydrodynamic forces during floods, and wind-induced vibrations during storms [1]. However, prolonged exposure to such operational stressors inevitably leads to progressive performance degradation, manifested through material fatigue, structural damage accumulation, and surface irregularities. These structural deteriorations not only compromise the load-bearing capacity but also adversely affect vehicular dynamics, potentially triggering catastrophic failure mechanisms that could result in incalculable socioeconomic consequences [2,3].

As of the end of 2023, China has built 1.0793 million highway bridges, an increase of 46,100 bridges compared with the previous year. In recent years, the growth rate of bridges has remained steady, with an average annual increase of 45,500 bridges. While maintaining an annual increase of several tens of thousands of new bridges, China is also tasked with managing and maintaining nearly one million existing bridges. Therefore, achieving efficient and safe operational management of this many bridges has become a pressing challenge. Indeed, many developed countries are facing the challenge of aging and inadequate infrastructure, coupled with limited financial resources for their maintenance. However, reliable bridge structural health-monitoring systems (BHMSs) offer the potential to transition from time-based inspection schedules to more cost-effective, condition-based maintenance strategies [4]. Currently, over 500 bridges in China are equipped with BHMSs, which play an increasingly significant role in bridge safety and lifespan prediction. By deploying a network of sensors on bridges, it is possible to continuously monitor environmental factors, load effects, and local and global structural responses [5]. The data provided by health-monitoring systems can be used to study the damage and performance degradation of in-service bridge structures and analyze and establish quantitative models for assessing the service safety and remaining lifespan of the structures, which is a critical research topic for improving the safety and durability of road bridges [6].

With the rapid development of Internet of Things (IoT) technology and Wireless Sensor Networks (WSNs), BHMSs are now capable of the real-time collection and transmission of various physical parameter data, such as structural status, strain, displacement, acceleration, and temperature, through the self-organizing communication of wireless sensors. Compared with traditional wired deployment methods, wireless sensors can significantly reduce installation costs, saving at least 50% of expenses [7]. For example, on the Bill Emerson Memorial Bridge, 84 wired accelerometers were used, with installation costs exceeding USD 15,000 per channel, half of which was attributed to cable wiring costs [8,9,10]. Mobile wireless sensors are sensor devices capable of moving flexibly in physical space and transmitting data via wireless communication technologies. These sensor systems exchange data with remote data acquisition and processing systems through built-in wireless network modules, enabling them to perform data-monitoring tasks in various dynamic environments [11]. Due to their mobility, flexibility, low-cost, and wireless characteristics, the application of HMWSs in bridge monitoring not only improves accuracy and efficiency but also reduces maintenance costs, enhances emergency response capabilities, and contributes to extending the lifespan of bridges.

This paper addresses the issues of the rational deployment and coverage enhancement of HMWSs, focusing on maximizing the coverage area and minimizing the movement distance of HMWSs. The Hiking Optimization Algorithm (HOA) [12] features a novel structure with few control parameters, demonstrating strong optimization performance in solving numerous numerical optimization and engineering problems. This paper proposes an improved HOA (GPTHOA) based on Good Point Set theory and t-distribution perturbation with heterogeneous degrees of freedom, which is then combined with the virtual force algorithm (VFA) to form the VF-GPTHOA. This method is applied to optimize the deployment of HMWSs for bridge monitoring in a bridge model (BM). The core innovations and contributions of this paper include the following:

1. Two strategies are adopted to improve the HOA and address the strong dependence on initial solutions and the lack of mechanisms to escape local optima in the HOA. First, the Good Point Set theory generates an evenly distributed initial population, enhancing the algorithm’s exploration and stability. Second, a t-distribution perturbation operator with heterogeneous degrees of freedom is introduced to perturb individuals randomly, and the Metropolis criterion is applied to accept poorer solutions with a certain probability, helping the algorithm escape local optima.
2. The VFA is introduced and combined with the GPTHOA to form the VF-GPTHOA, improving the algorithm’s convergence speed and accuracy in solving the HMWS deployment optimization problem in the band-like deployment environment of bridges. Once optimization is completed, the GPTHOA determines the optimal paths for sensor nodes to minimize energy consumption.
3. The performance of the GPTHOA is tested on the CEC2022 benchmark test functions. In the bridge model (BM), the VF-GPTHOA is applied to optimize the deployment of HMWS nodes. Simulation results demonstrate that the proposed VF-GPTHOA outperforms other algorithms in multiple performance metrics.

## 2. Related Works

Experts and scholars have primarily focused on virtual force algorithms and swarm intelligence optimization algorithms to improve WSN coverage and service quality. Especially in recent years, with the rapid development of swarm intelligence optimization algorithms, multiple approaches have been developed to address complex problems such as WSN coverage optimization.

### 2.1. Virtual Force Algorithm

The virtual force algorithm draws inspiration from the principles of mechanics in physics, treating the interactions between sensor nodes as manifestations of virtual forces. By properly adjusting the magnitude and direction of attraction and repulsion forces, nodes can autonomously adjust their positions based on the current network conditions, thereby reducing overlap between nodes and achieving better coverage [13]. Fei et al. [14] defined the attraction of grid points to sensor nodes and their thresholds and, by incorporating the virtual force between nodes and obstacles, improved the traditional algorithm. Simulation results demonstrate that this method exhibits superior performance in terms of coverage, energy consumption, redundancy, and connectivity. Kiani et al. [15] investigated the relationship between the repulsive force of adjacent nodes and the attractive force of target points by using the minimum number of sensor nodes. They proposed a greedy algorithm that optimizes network coverage performance through this strategy. To reduce coverage holes and achieve uniform distribution, Qin et al. [16] divided the monitoring area into a cellular grid, using heterogeneous sensor nodes with different sensing radii. They analyzed the virtual forces between nodes based on their distances and constructed a bipartite graph between the cellular grid and sensor nodes. Subsequently, they employed the vampire bat algorithm to match these two components, enhancing coverage and optimizing energy. Chao et al. [17] optimized the distribution of sensor nodes by setting two types of virtual forces. The first type of virtual force acts between sensor nodes, while the second type is the force generated between concentric rings after dividing the network into multiple equal-width concentric rings. Compared with other methods, this strategy shows significant advantages in improving coverage and extending network lifetime. To balance coverage and network lifetime in WSNs, Qi et al. [18] derived the minimum required number of sensors through mathematical analysis and enhanced WSNs’ coverage capability by combining the traditional virtual force algorithm with the Voronoi diagram (VFVG) while also reducing the movement distance of sensor nodes. Furthermore, based on the trust relationships between nodes, they proposed a novel trust-driven topology construction and maintenance method, which effectively extends the network’s lifespan.

N. Boufares et al. [19] extended the original virtual force algorithm (VFA) to three dimensions, resulting in the Three-Dimensional Virtual Force Algorithm (3DVFA), suitable for three-dimensional environments. Based on the dodecahedral tessellation principle, the 3DVFA defines distance thresholds and restricts the movement range of nodes, thereby improving network coverage and connectivity. However, the 3DVFA overlooks the feasibility of truncated octahedra in stacking three-dimensional regions, leading to inadequate coverage performance. Meanwhile, their team [20] proposed a distributed deployment algorithm based on an improved virtual force strategy. The algorithm ensures network connectivity, eliminates node oscillations, and reduces network energy consumption by deploying mobile sensor nodes within the three-dimensional region. Luo et al. [21] proposed the Three-Dimensional Virtual Force Coverage Algorithm (3D-VFCA), which enables the redeployment of underwater WSN nodes to restore higher coverage.

Although the virtual force algorithm plays a positive role in improving coverage performance, it is vulnerable to oscillation because it only considers the virtual force of a single node and ignores the layout of the whole network, which limits the coverage optimization effect of WSNs to a certain extent.

### 2.2. Intelligent Optimization Algorithm

Swarm intelligence-based coverage optimization algorithms do not rely on the problem itself and offer advantages such as scalability, robustness, and efficiency [11,22]. Inspired by the predation behavior of army ants, a novel Army Ant Search Optimization Algorithm was proposed in [23] and demonstrated superior performance in both local search and global exploration, showing to be particularly effective in optimizing the positions of sensor nodes. Wang et al. [24] improved the Gray Wolf Optimization (GWO) algorithm and introduced a new method for addressing perception blind spots and calculating three-dimensional surface coverage areas. This approach not only reduces network costs but also achieves broader coverage for Wireless Sensor Networks (WSNs). To address deployment issues in complex environments, the team optimized the GWO position update formula by incorporating a nonlinear convergence factor and elite strategy, thus improving the convergence accuracy of the algorithm. In WSN deployment, the improved algorithm achieved greater coverage with the minimum number of sensor nodes [25]. Wang et al. [26] proposed the Adaptive Multi-Strategy Artificial Bee Colony (SaMABC) algorithm to address the coverage optimization problem in Wireless Sensor Networks (WSNs). The algorithm introduces a new adaptive selection mechanism and search strategy, forming a multi-strategy pool. Additionally, by combining simulated annealing and dynamically adjusting the search step size, the overall performance of the algorithm is further improved. Simulation experiments demonstrate that this algorithm outperforms traditional methods across various deployment scenarios. Chowdhury et al. [27] combined Voronoi diagrams, the Firefly Algorithm, and k-means clustering to propose a novel energy-efficient coverage technique that effectively enhances coverage and reduces network energy consumption. Deepa et al. [28] introduced an enhanced whale optimization algorithm based on Levy flights to achieve coverage optimization. Syed et al. [29] proposed the Weighted Tunicate Swarm Algorithm based on weighted distance position updates, effectively balancing WSN coverage efficiency and energy consumption. In [30], the authors presented the Improved Gravitational Search Coverage Control Algorithm (IGSCCA), aimed at improving coverage and node utilization in WSNs while minimizing overlap between sensor nodes. The algorithm combines the virtual force algorithm with the gravity disturbance of grid points in an improved gravitational search algorithm during the coverage enhancement phase and introduces a sleep strategy based on safe sets during the node sleep phase, thereby optimizing network performance. Yao et al. [31] proposed the Virtual Force-Guided Improved Moth-Flame Optimization (VF-IMFO) algorithm. This algorithm enhances the moth search process by improving the position update formula, incorporating an adaptive inertia weight strategy, and using the collective force of nodes as a disturbance factor, thus effectively optimizing the search path of the moths. This method not only improves network coverage but also reduces the displacement of sensor nodes. Cao et al. [32] improved the original Swarm Spider Optimization (SSO) algorithm by incorporating neighborhood search, global search, and matching radius techniques to enhance the algorithm’s optimization performance. In addressing the coverage enhancement issue of heterogeneous WSNs, this algorithm significantly improves network coverage, thereby effectively reducing the number of coverage blind spots.

Biomimetic mechanism-based swarm intelligence optimization algorithms perform global optimal solution search for complex optimization problems by simulating the cooperative behavior of biological populations. These algorithms demonstrate significant advantages in multidimensional search and efficient parallel computation in the field of area coverage optimization. However, such algorithms still face technical bottlenecks in terms of parameter configuration and sensitivity to initial population distribution.

In summary, the virtual force algorithm drives nodes to rapidly respond to coverage holes through local force fields, demonstrating high timeliness in dynamic adjustments. However, its single-node force guidance mechanism is prone to inducing oscillations in complex and elongated bridge structures, leading to coverage fluctuations. In contrast, swarm intelligence optimization algorithms perform global optimal search through population collaboration, but their performance is highly sensitive to parameter settings and initial distribution, making them susceptible to premature convergence in complex and elongated bridge structures.

Relevant studies indicate that combining swarm intelligence optimization algorithms with virtual force algorithms can significantly improve the coverage and overall performance of WSNs [33]. Additionally, it is important to note, in accordance with the “No Free Lunch Theorem” [34], that no optimization algorithm performs best for all engineering problems. Given that the HOA has advantages such as fewer parameters and strong optimization capability, this paper proposes the VF-GPTHOA and successfully applies it to the key technology of HMWS coverage optimization in bridge monitoring.

## 3. Models and Definitions

### 3.1. Bridge Model (BM)

**Figure 1:**
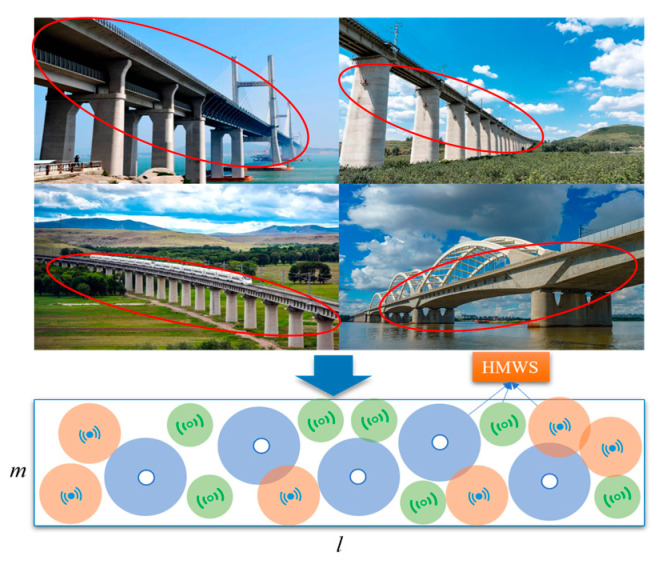
The bridge monitoring model (Different colors and symbols represent different types of sensors).

The WSN nodes required for bridge structural health monitoring are typically deployed at key locations on the bridge after its construction, based on specific monitoring needs and objectives. By deploying various types of wireless sensor nodes, the precise monitoring of the bridge’s health status can be achieved, enabling the timely detection of potential structural issues such as cracks, corrosion, and overloads. This provides a scientific basis for the maintenance and repair of the bridge, extending its service life and ensuring traffic safety [5,35]. HMWSs can adaptively adjust the deployment location according to the monitoring requirements and structural changes of the bridge so as to focus on monitoring the key parts of the bridge, especially in different areas of the bridge, or when abnormalities occur, it can quickly adjust the location to optimize the monitoring coverage. As shown in Figure 1, in order to quickly validate the theoretical method proposed in this paper, the BM treats the entire bridge as a two-dimensional plane with dimensions of l×m, where m represents the width of the bridge deck, l represents its length, and l≫m. The HMWS is deployed in this model, and the base station can remotely control the HMWS through instructions.

### 3.2. Coverage Model

Currently, there are two primary sensing models in research on the coverage theory of WSNs: the Boolean sensing model and the probabilistic sensing model. The Boolean sensing model defines the coverage area of sensors through geometric relationships. Due to its simplicity, intuitiveness, and high computational efficiency, it has become a commonly used model for verifying the feasibility and effectiveness of proposed methods. Therefore, this paper adopts the Boolean sensing model to validate the coverage optimization method.

**Figure 2:**
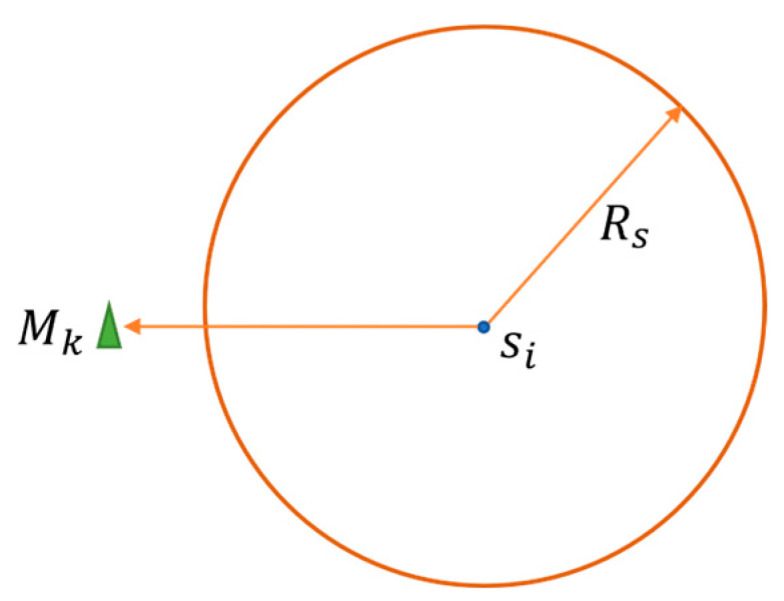
Boolean sensing model.

As shown in Figure 2, $M_{k}$ is the target monitoring point. The sensing range of a node $s_{i}$ is a circular area with $s_{i}$ as the center and $R_{s}$ as the radius (determined by the hardware characteristics of the node). Only the points within this area can be sensed and covered by the node. The communication radius $R_{c}$ of nodes generally satisfies $R_{c}\geq 2R_{s}$. For ease of calculation, the monitoring area is divided into m×l grid points. Let us suppose that the coordinate of the target monitoring point (that is, a grid point) $M_{k}$ is $\left(x_{k}{,y}_{k}\right),k=1,2,\cdots ,m\times l$, and the coordinate of node $s_{i}$ is $\left(x_{i}{,y}_{i}\right),i=1,2,\cdots N$. Then, the mathematical expression of the Boolean perception model is shown in Equation (1).

(1) $$C_{\left(s_{i},M_{k}\right)}=\left\{\begin{array}{c} 1,\;d_{\left(s_{i},M_{k}\right)}\leq R_{s}\;\\ 0,d_{\left(s_{i},M_{k}\right)}\geq R_{s} \end{array}\right.,$$

where $C_{\left(s_{i},M_{k}\right)}$ represents the sensing probability of node $s_{i}$ to target monitoring point $M_{k}$ and $d_{\left(s_{i},M_{k}\right)}$ represents the Euclidean distance between two nodes, which is calculated by Equation (2).

(2) $$d_{\left(s_{i},M_{k}\right)}=\sqrt{{\left(x_{i}-x_{k}\right)}^{2}+{\left(y_{i}-y_{k}\right)}^{2}}$$

### 3.3. Definitions

**Definition 1** (coverage rate (CR)). *In the Boolean sensing model, when a target monitoring point* $M_{k}$ *is sensed by the sensor nodes set* S*, the point is considered to be coverable. A monitoring point is not covered if none of the sensor nodes in set *S *detect it. Therefore, as shown in Equation (3), the CR is defined as the ratio of the total number of grid points covered to the total number of grid points in the BM.*

(3) $$CR=\frac{\sum_{k=1}^{m\times l}C_{\left(S,M_{k}\right)}}{m\times l}$$

**Definition 2** (coverage efficiency (CE)). *CE measures the effective utilization of sensor nodes. Larger values of CE result in a more uniform distribution of nodes and lower redundancy. As shown in Equation (4), CE is defined as the ratio of the total area of the monitoring area covered by all nodes to the total sensing area that all nodes should have covered.*

(4) $$CE=\frac{CR\times m\times l}{N\times \pi \times R_{s}^{2}}$$

**Definition 3** (overlap ratio (OR)). *The OR is defined as the ratio of the total number of monitoring points covered repeatedly to the total number of grid points in the BM. When two or more sensor nodes in node set S cover monitoring point $M_{h}$, then the grid point is repeatedly covered. A smaller OR represents a smaller overlap area and, at the same time, fewer redundant nodes. The OR of a monitoring point can be expressed by Equation (5).*

(5) $$P_{\left(s_{i},s_{j},M_{h}\right)}=\left\{\begin{array}{c} \;1,ifd_{\left(s_{i},M_{h}\right)}\leq R_{s}\;and\;d_{\left(s_{j},M_{h}\right)}\leq R_{s}\;\\ 0,otherwise\; \end{array}\right.$$

*When a monitoring point* $M_{h}$ *is covered by both nodes* $s_{i}$ *and* $s_{j}$ *(* i,j∈{1,2,⋯,N} *), it means that* $M_{h}$ *is covered repeatedly. The total probability of the repeated coverage of monitoring points in the BM of the whole monitoring area can be calculated according to Equation (6).*

(6) $$P_{\left(S,M_{h}\right)}=1-\prod_{i=1,j>i}^{N}\left(1-P_{\left(s_{i},{s_{j},M}_{h}\right)}\right)$$

*Therefore, the OR can be expressed by Equation (7).*

(7) $$OR=\frac{\sum_{k=1}^{m\times l}P_{\left(S,M_{h}\right)}}{m\times l}$$

**Definition 4** (Average Moving Distance (AMD)). *Mobile sensor nodes can be re-deployed autonomously by moving, and the energy consumption in this process is mainly generated by node movement, which is mainly determined by the moving distance of the node [36]. Therefore, in this paper, the moving distance is used as a measure of node energy consumption. In the process of re-autonomous deployment, the moving distance of the sensor node $s_{i}$ is $d_{i}$; then, the average moving distance of all nodes in the network can be expressed by Equation (8).*

(8) $$AMD=\frac{1}{N}\sum_{i=1}^{N}d_{i}$$

### 3.4. The Theory of Optimal Coverage

**Figure 3:**
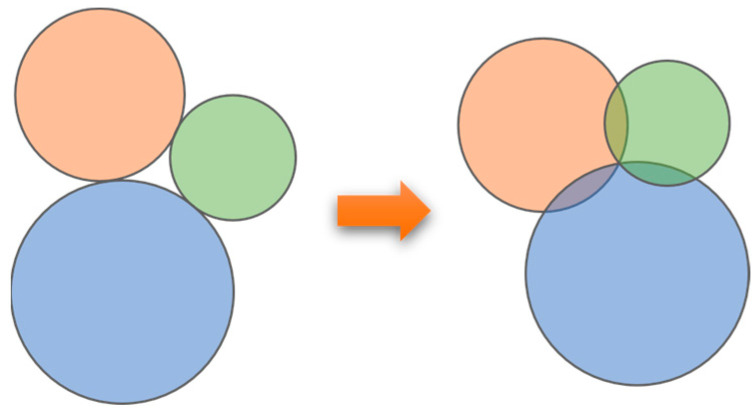
Diagram of optimal deployment.

The minimum number of sensor nodes to achieve complete coverage of the monitoring area is called optimal coverage [37]. As shown in Figure 3, when there is no overlap in the sensing range of the sensors, this scheme theoretically maximizes the sensing coverage of the sensor nodes. However, coverage gaps exist between any three nodes. To achieve complete coverage, the sensing ranges of any pair of adjacent sensor nodes should overlap by a constant amount, and the sensing ranges of any three adjacent sensor nodes should intersect at a single point. This deployment strategy allows the minimum number of sensor nodes to achieve complete coverage.

## 4. Hiking Optimization Algorithm (HOA)

The HOA was proposed by Sunday O. Oladejo et al. [12] in 2024, inspired by the experience of hikers attempting to ascend mountain ranges, hills, or rock peaks. During the hiking process, hikers consciously or unconsciously consider the steepness of the terrain, avoiding steep areas and narrow paths to maintain their hiking speed. The HOA simulates the characteristics of a hiking expedition where the ultimate goal is to reach the summit, with the environment being a rugged terrain that includes both local maxima and the global maximum. The situation experienced by hikers during the ascent is analogous to an agent trying to find a local or global optimum in an optimization problem.

The mathematical basis of the HOA is Tobler’s Hiking Function (THF), proposed by famous Swiss geographer Waldo Tobler in 1993, also known as Tobler’s walking equation [38]. This model describes the relationship between human movement speed and terrain slope, and its specific mathematical form is shown in Equation (9).

(9) $$w_{i,t}=6e^{-3.5\left|s_{i,t}+0.05\right|}$$

where $w_{i,t}$ represents the walking speed (in km/h) of traveler i at time t and $s_{i,t}$ is the slope of the terrain, which is given by Equation (10).

(10) $$s_{i,t}=\frac{dh}{dx}=\tan\theta_{i,t}$$

where dh and dx are the elevation and distance, respectively, and $\theta_{i,t}$ is the slope of the terrain or trail, and its value ranges within [0, 50°].

**Figure 4:**
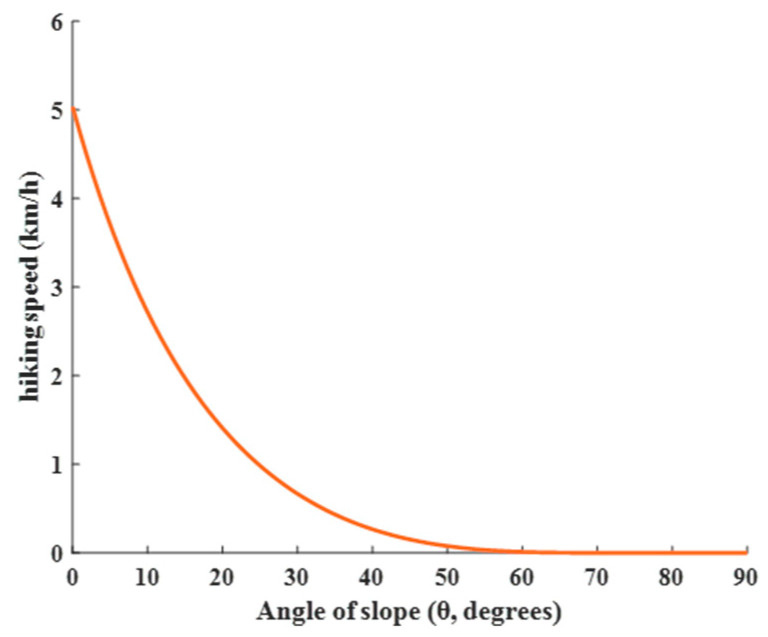
Image of THF.

Figure 4 gives an image of the THF, which indicates that hikers move slower as the slope of the terrain increases. The negative exponential term in the equation captures this relationship. It should be noted that this function provides an estimate of walking speed and is based on the averaging assumption. Individual differences, physical fitness, road conditions, and other factors may affect the actual walking speed. Nonetheless, the Tobler walking function is widely used in Geographic Information Systems (GISs) and related fields to model pedestrian movement and accessibility analysis.

The HOA uses the hiker group’s social thinking and the individual hiker’s personal cognition to update the location. The current speed update formula of the traveler in the HOA is as follows:

(11) $$w_{i,t}=w_{i,t-1}+r_{i,t}(\beta_{best}-\alpha_{i,t}\beta_{i,t})$$

where $r_{i,t}$ is a random number in [0, 1], $w_{i,t}$ and $w_{i,t-1}$ represent the current and initial velocities of traveler i, $\beta_{best}$ is the position of the leader, $\beta_{i,t}$ is the position of traveler i at time t, and $\alpha_{i,t}$ is the sweep factor (SF) of traveler i in the range [1, 3], which ensures that the traveler does not stray too far from the leader, clearly sees the direction of the leader, and receives the signal from the leader.

By considering the velocity of the traveler in Equation (9), the position update $\beta_{i,t+1}$ of traveler i is given by Equation (12).

(12) $$\beta_{i,t+1}=\beta_{i,t}+w_{i,t}$$

Like most metaheuristic optimization algorithms, the HOA uses a random initialization technique to set the initial positions of the agents. The exploration and exploitation tendencies of the HOA are influenced by the SF, which affects the distance between the leader and other hikers. The slope of the terrain or path impacts the hiker’s speed. The HOA tends to favor the exploitation phase as the SF range increases, while a decrease in the SF range promotes the exploration phase. Additionally, a decrease in the slope leads the HOA to lean towards the exploitation phase.

**Figure 5:**
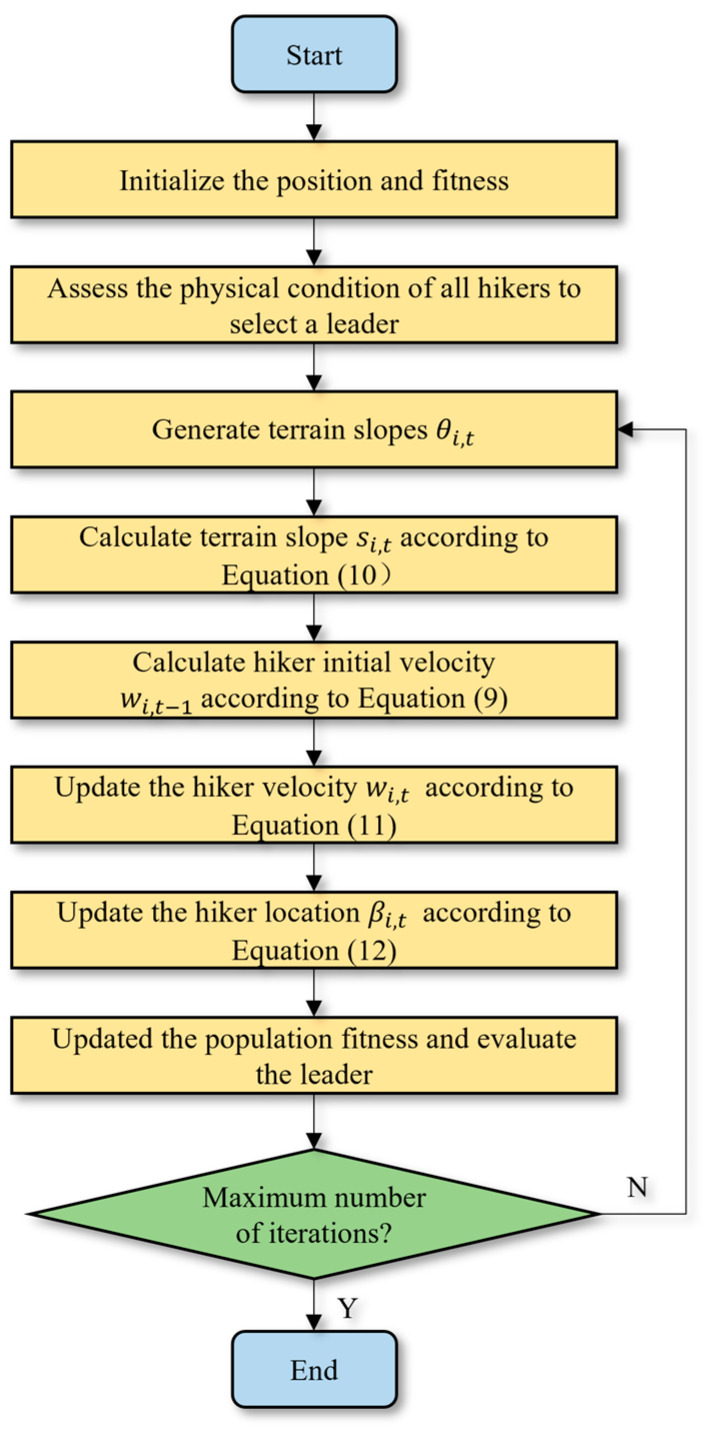
The flowchart of the HOA.

The HOA is a global optimization algorithm that fully leverages hikers’ experience when traversing mountains and ascending peaks. At the beginning of the hike, the initial positions of the hikers are determined, and the THF is used to estimate the new positions of the travelers after a given time. The physical condition of all hikers is evaluated, and the fittest individual becomes the leader. In each iteration, the leader’s position is reassessed to ensure that the most suitable hiker is designated as the leader. Figure 5 presents the flowchart of the HOA.

## 5. HMWS Deployment Optimization Algorithm Based on GPTHOA

To improve the performance of HMWSs in secondary autonomous deployment optimization within BMs, this paper proposes a Hiking Optimization Algorithm based on the Good Point Set theory and heterogeneous-degree-of-freedom t-distribution perturbation (GPTHOA). The GPTHOA significantly enhances stability and the ability to escape local optima compared with the original HOA. Furthermore, this paper analyzes the virtual forces between HMWS nodes in the monitoring area. It uses the total virtual force at each node as an interference factor for updating the hiker’s search position. This guides the nodes to move towards the direction of coverage gaps, thus completing the secondary autonomous deployment optimization.

### 5.1. Initializing the Population by the Good Point Set

Using the Good Point Set strategy [39] to improve the population random initialization of the HOA can improve the convergence speed and stability of the HOA. The Good Point Set is defined as follows:

- Set $G_{s}$ is an s unit cube in European space, namely, $x\in G_{s},x=(x_{1},x_{2},\cdots x_{s})$, where $0\leq x_{i}\leq 1,i=1,2,\cdots ,s$.
- Set $G_{s}$ contains a set of n points $P_{n}\left(k\right)=\{x_{1}^{\left(n\right)}\left(k\right),x_{2}^{\left(n\right)}\left(k\right),\cdots ,x_{s}^{\left(n\right)}\left(k\right),1\leq k\leq n$, where $0\leq x_{i}^{\left(n\right)}\left(k\right)\leq 1,i=1,2,\cdots ,s$.
- For any given point $r=(r_{1},r_{2},\cdots {,r}_{s})$ in $G_{s}$, let $N_{n}\left(r\right)=N_{n}\left(r_{1},r_{2},\cdots {,r}_{s}\right)$ denote the number of points in $P_{n}\left(k\right)$ that satisfy the system of inequalities in (13).
  (13) $$\left\{\begin{array}{c} 0\leq x_{i}^{\left(n\right)}\left(k\right)\leq r,\;i=1,2,\cdots ,s\\ \Phi \left(n\right)=Sup\left|\frac{N_{n}\left(r\right)}{n}-\left|r\right|\right|\; \end{array}\right.$$
  where $\left|r\right|=r_{1}r_{2}\cdots r_{s}$ and point set $P_{n}\left(k\right)$ is considered to have a deviation $\Phi \left(n\right)$. If, for any n, $\Phi \left(n\right)=O(1)$, then $P_{n}\left(k\right)$ is considered to be uniformly distributed on $G_{s}$ with a deviation of $\Phi \left(n\right)$.
- Let $r\in G_{s}$ and the deviation $\Phi \left(n\right)$ of $P_{n}\left(k\right)=\left\{\left\{r_{1}\cdot k,\cdots ,r_{s}\cdot k\right\}|k=1,2,\cdots ,n\right\}$ satisfy $\Phi \left(n\right)=C(r,\varepsilon )n^{\left(-1+\varepsilon \right)}$, where C(r,ε) is a constant that depends only onr and ε (with ε being an arbitrarily small positive number). In this case, $P_{n}\left(k\right)$ is called a Good Point Set, and r is called a good point.
- Let $r_{k}=2\cos\left(\frac{2\pi k}{p}\right),1\leq k\leq s$, where p is the smallest prime number that satisfies $\frac{p-s}{2}\geq t$, or let $r_{k}=e^{k}$, 1≤k≤s. In this case, r is a good point.

**Figure 6:**
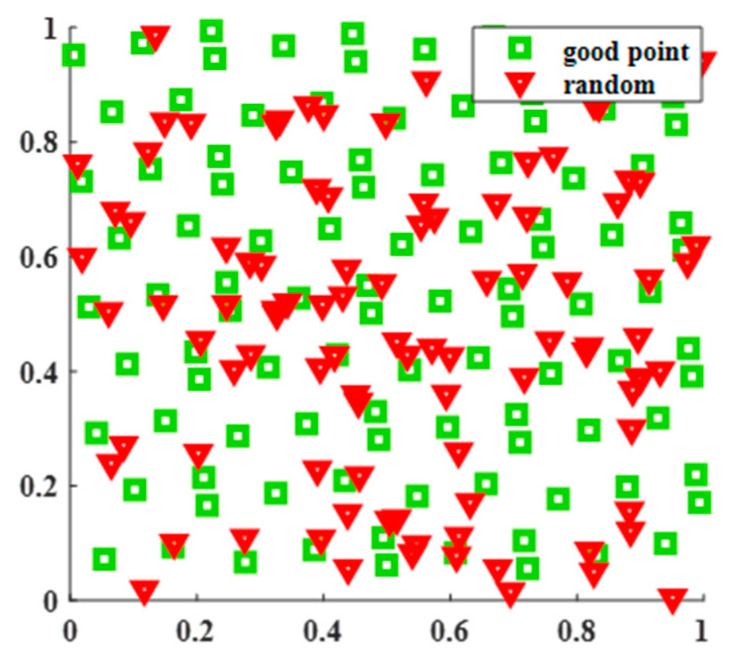
Comparison of Good Point Set and random initialization.

As shown in Figure 6, assuming a population size of 100, when initializing the population in a two-dimensional space, it can be visually observed that the population distribution initialized by using the Good Point Set strategy is more uniform. Therefore, this paper completes population initialization by mapping Good Point Set $P_{n}\left(k\right)$ to the feasible region of the population, and the specific operation can be expressed as in (14).

(14) $$\beta_{i,t}=\phi_{j}^{1}{+P}_{n}\left(k\right)(\phi_{j}^{2}-\phi_{j}^{1})$$

where $\phi_{j}^{1}$ and $\phi_{j}^{2}$ denote the lower and upper bounds of the jth dimensional decision variable in the optimization problem, respectively.

### 5.2. Heterogeneous-Degree-of-Freedom t-Distributed Perturbation

The standard HOA lacks a mutation mechanism when performing a global search and can easily fall into a locally optimal solution. This paper introduces the t-distribution perturbation operator with heterogeneous degrees of freedom to randomly perturb the individuals, which endows the algorithm with diversity and stronger exploration ability. The proposed heterogeneous-degree-of-freedom t-distribution mutation perturbation formula is shown in (15).

(15) $$X_{i}^{t+1}=\frac{t}{iter\_max}\cdot X_{trnd\left(10\right)}^{t}+(1-\frac{t}{iter\_max})\cdot X_{trnd\left(2\right)}^{t}$$

where $trnd\left(10\right)$ is the t-distribution with 10 degrees of freedom and $trnd\left(2\right)$ is the t-distribution with 2 degrees of freedom. The selection of 2 degrees of freedom represents the minimum effective configuration (as variance becomes undefined at 1 degree of freedom), while that of 10 degrees of freedom approximates a normal distribution (which essentially coincides with the Gaussian distribution at 30 degrees of freedom). This parameter design ensures significant divergence between the two distributions while preventing computational instability caused by extreme parameter selection.

**Figure 7:**
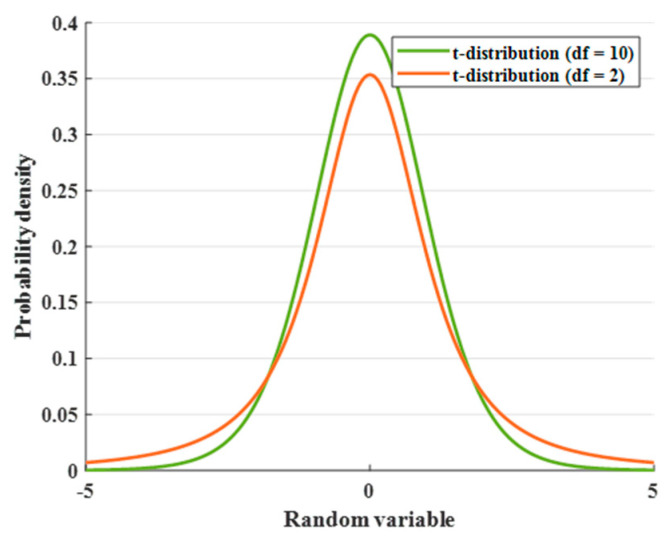
Probability density plot of t-distribution.

Figure 7 illustrates the t-distribution probability plots for different degrees of freedom. In the early stage of algorithm operation, $trnd\left(2\right)$ plays a leading role, which makes the algorithm stable in global exploration and avoids falling into local optimal solutions. $trnd\left(10\right)$ plays a leading role in the late iteration to achieve accurate local search and improve the optimization accuracy of the algorithm.

After the new solution is generated by using t-distribution disturbance with different degrees of freedom, the simulated annealing algorithm is referred to according to the Metropolis criterion [40]. The worse solution is accepted with a certain probability to jump out of the local optimal value, to make up for the algorithm defects of the HOA. The parameters of the Metropolis criterion are set as initial temperature $T_{0}=100$ and cooling coefficient α=0.9. The $T_{0}=100$ setting ensures adequate exploration capability in the solution space. The cooling rate of α=0.9 facilitates a smooth transition from global exploration to local exploitation. This parameter combination effectively balances the algorithm’s exploration–exploitation trade-off, significantly mitigating the risk of premature convergence.

**Figure 8:**
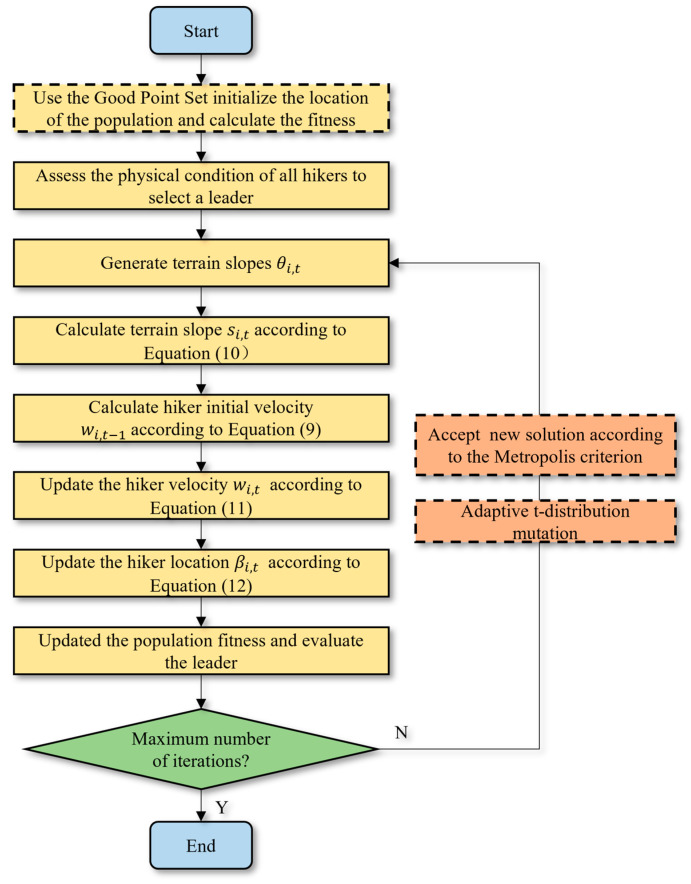
The flowchart of the GPTHOA.

The improved HOA in this paper is named GPTHOA, and its flow chart is shown in Figure 8.

### 5.3. Performance Test of GPTHOA

To test the performance of the GPTHOA in terms of solution accuracy, convergence speed, and stability, the GPTHOA was compared with the HOA, the SSOA [41], and LCA [42,43] on the benchmark function CEC2022. Table 1 presents the relevant information on the test functions. The population size was set to 100, and the number of iterations was set to 500, with dimensions of 10 and 20. To eliminate randomness, 30 experiments were conducted to ensure accuracy. Table 2 shows the mean, maximum, minimum, and Friedman mean of the optimal solutions obtained by the four algorithms in different dimensions, where the best values for each evaluation criterion are in bold.

**Figure 9:**
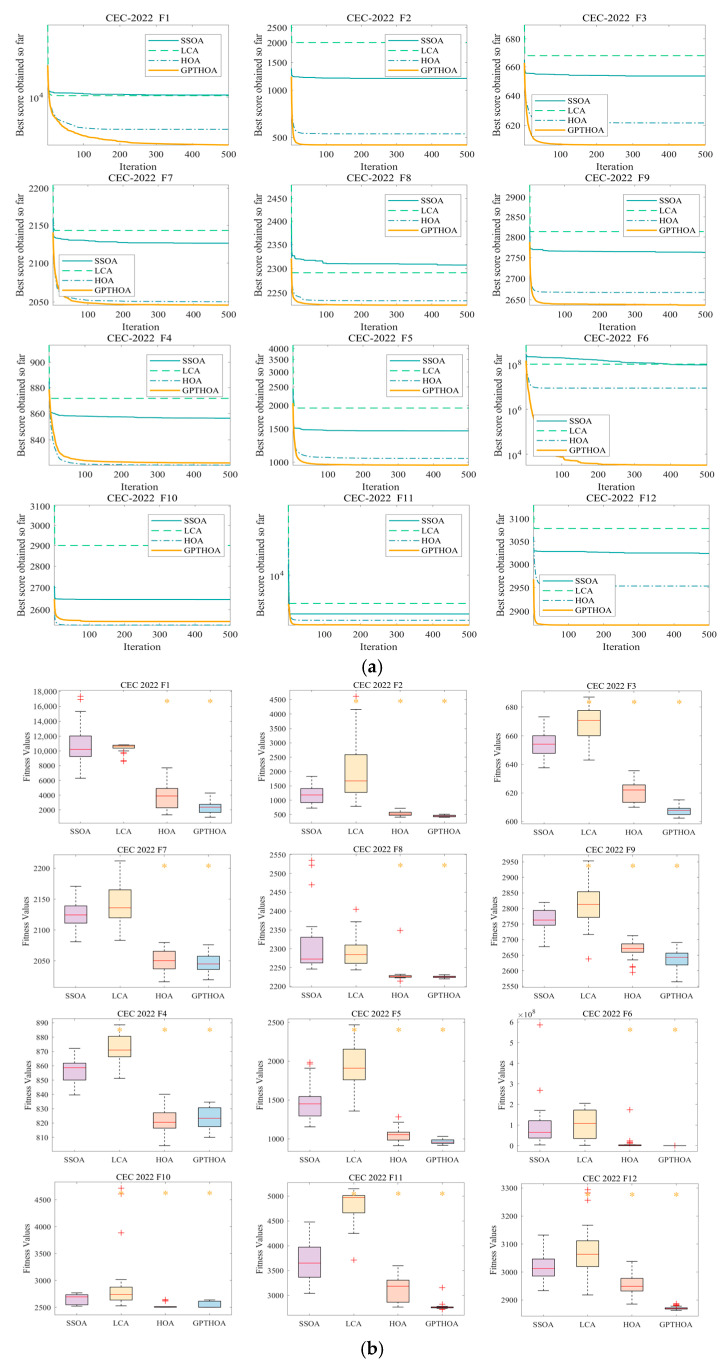
Optimizing CEC2022 in 10 dimensions. (**a**) Convergence process and (**b**) box plots (red plus signs and orange asterisks indicate low and high outliers, respectively).

**Figure 10:**
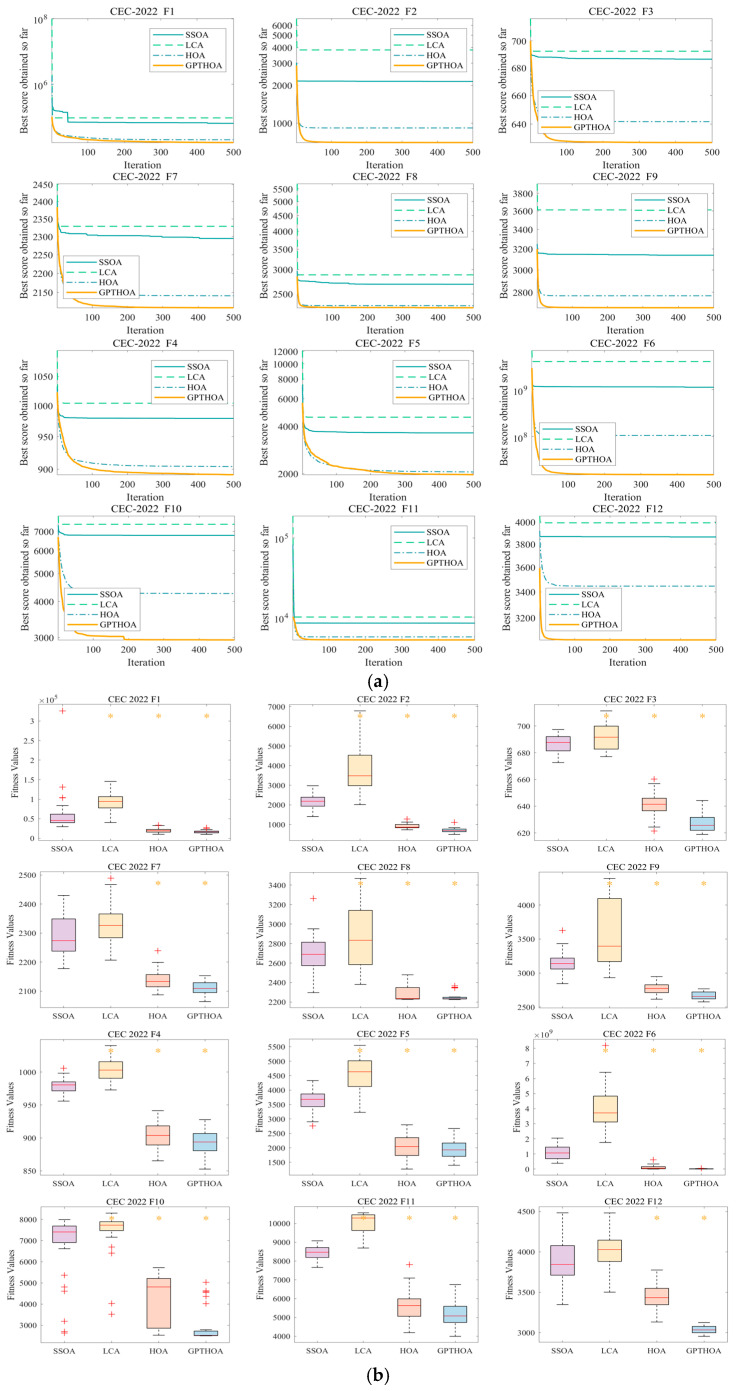
Optimizing CEC2022 in 20 dimensions. (**a**) Convergence process and (**b**) box plots (red plus signs and orange asterisks indicate low and high outliers, respectively).

Table 2 presents the optimization results of the SSOA, LCA, the HOA, and the GPTHOA on the benchmark function CEC2022 in different dimensions. Whether in the 10-dimensional or 20-dimensional case, the proposed GPTHOA significantly outperforms other algorithms in overall performance. Moreover, in the Friedman test, the GPTHOA achieves smaller mean values in different dimensions, indicating that the GPTHOA exhibits greater stability and consistency across multiple experiments. This is due to the GPTHOA’s use of an optimal point set to initialize the population, ensuring that the initial population includes individuals close to the optimal solution. The GPTHOA does not need to explore the entire search space from scratch, reducing the time required for search space exploration. Additionally, the t-distribution perturbation and simulated annealing operations help the algorithm escape local optima, enhancing its diversity. To visually observe the optimization performance of the algorithms, Figure 9 and Figure 10 show the four algorithms’ convergence processes and box plots while optimizing CEC2022 in different dimensions. The GPTHOA performs the best in convergence accuracy, speed, and stability compared with the other three algorithms. Whether in the 10-dimensional or 20-dimensional case, the GPTHOA demonstrates stronger optimization capabilities on over 10 functions, with the fastest and most stable convergence speed.

In summary, the GPTHOA’s advantages in convergence speed and optimization performance allow it to be applied to the optimization of HMWS deployment for bridge monitoring.

### 5.4. Virtual Force-Guided Coverage Optimization

Given that the virtual force algorithm (VFA) [13] has high convergence speed and can effectively overcome the issues of slow convergence and susceptibility to local optima in swarm intelligence optimization algorithms, this paper introduces the virtual force mechanism into the algorithm’s search process. The virtual force consists of three components: first, the virtual force between adjacent sensor nodes; second, the virtual attraction force for uncovered grid points; and third, the virtual repulsion force at the boundaries. The virtual resultant force $\;F_{i}$, composed of these three forces, acts as a disturbance factor and guides the nodes to move toward the coverage holes. Under the influence of the virtual resultant force $F_{i}$, the sensor $s_{i}\;$moves from its original position (${x_{iold},y}_{iold}$) to the optimal position (${x_{inew},y}_{inew}$), and the expression for the optimal position can be represented by Equation (16).

(16) $$\left\{\begin{array}{c} x_{inew}=x_{iold}+\frac{F_{ix}}{F_{i}}\times Mov\_step\times e^{-\frac{1}{F_{i}}}\\ y_{inew}=y_{iold}+\frac{F_{iy}}{F_{i}}\times Mov\_step\times e^{-\frac{1}{F_{i}}} \end{array}\right.$$

where $F_{ix}$ and $F_{iy}$ represent the projections of the resultant force $F_{i}$ on the *x*-axis and *y*-axis, respectively, and *Mov*_*step* represents the step size of the sensor node’s movement in each iteration. In the early stages of the algorithm, a larger step size is set to accelerate convergence. As the number of iterations increases, a smaller step size is adopted to prevent oscillation in the algorithm. The virtual movement step size gradually decreases throughout the iteration process, and its variation can be expressed by Equation (17).

(17) $$Mov\_step=Max\_st-(Max\_st-Min\_st)\times \frac{t}{iter\_max}\times e^{-\frac{t}{iter\_max}}$$

where *Max*_*st* and *Min*_*st* represent the maximum and minimum movement step sizes, with values of 2 and 0.1, respectively. The parameter selection of a maximum step size of 2 and a minimum step size of 0.1 fundamentally reflects a trade-off between exploratory capacity (extensive spatial coverage) and exploitation precision (fine-grained adjustments) while incorporating sensor physical constraints and algorithmic convergence requirements.

The GPTHOA is combined with the VFA to form an effective bridge monitoring wireless sensor node deployment optimization strategy, called the Virtual Force-Guided GPTHOA (VF-GPTHOA). Table 3 shows the correspondence between HMWS deployment optimization and the VF-GPTHOA. Each hiker corresponds to a deployment scheme, with the hiker’s position and fitness value corresponding to the sensor node’s position and network coverage rate (CR), respectively.

The VF-GPTHOA is used to solve the bounded constrained optimization problem of maximizing the coverage of BWSN nodes, and its main steps are as follows (the pseudocode is shown in Algorithm 1):

Step 1: Initialize the BM’s size, the number of sensor nodes, the coverage range, the relevant parameters of the VF-GPTHOA, and the maximum number of iterations.

Step 2: Initialize the deployment solution set by using Equation (14), and calculate each individual’s fitness value by using Equation (3). It is important to note the conversion between the maximization problem and the minimization problem.

Step 3: The VF-GPTHOA begins optimization. Calculate the hiker’s initial velocity by using Equation (9), update the hiker’s velocity by using Equation (11), and update the hiker’s position by using Equation (12).

Step 4: After perturbation generates a new solution by using Equation (15), accept the worse solution with a certain probability based on the Metropolis criterion to avoid getting trapped in local optima.

Step 5: For hikers with poor fitness values, use the virtual force represented by Equations (16) and (17) to perturb and update their position.

Step 6: Update the hiker’s fitness value, and save the best individual, i.e., the leader.

Step 7: Check if the iteration count has reached the upper limit. If not, return to Step 3; if yes, proceed to Step 8.

Step 8: Output the leader’s information, including the leader’s position and final fitness value.
**Algorithm 1** VF-GPTHOA**Require:** *Sensor node set:* $S=\{s_{1},s_{2},\cdots ,s_{i},\cdots ,s_{N}\}$*; Perceived distance: *$R_{s}$*; Total number of hikers: N and Maximum iterations: iter_max.* **Ensure:** *Sensor nodes’ location and CR.* **1:**  *Initialize the set of deployment scheme using (14).* **2:**  ***for***
i=1→N
***do**        //Iterate over all hikers (population individuals)* **3:**    *Evaluate a hiker’s fitness using (3).* **4:**  ***end for*** **5:**  *Generate initial optimal deployment schemes:* $\beta_{best}$   *//Record the current optimal deployment scheme*
$\beta_{best}$*t (highest fitness)* **6:**  ***while***
t<iter_max            *//Main optimization loop (iteration number control)* **7:**    ***for***
i=1→N ***do**           //An independent search is performed for each hiker* **8:**     *Extract initial position of hiker i:* $\beta_{i,t}$ **9:**     *Determine terrain angle of elevation:* $\theta_{i,t}$ **10:**    *Compute the slope*
$s_{i,t}$
*using (10).* **11:**    *Compute the initial hiking velocity*
$w_{i,t-1}$
*using (11).* **12:**    *Determine the actual velocity*
$w_{i,t}$
*using (11).* **13:**    *Update the hiker’s position using (12).* **14:**    ***end for*** **15:**    *Generate new deployment scheme by (15).* **16:**    *Decide whether to accept the new deployment scheme by Metropolis criterion.* **17:**    *Update the deployment schemes with poor CR by (16) and (17).* **18:**    *Update*
$\beta_{best}$
*if there is a better solution.   //Retain the historical best solution* **19:**    t=t+1      //Iterate counter increment **20:**  ***end while*** **21:**  ***return*** $\beta_{best}\&\;CR$       //Return the optimal deployment and its coverage

## 6. Simulation Results

To verify the comprehensive performance of the VF-GPTHOA proposed in this paper, the experiment uses Matlab R2022b as the simulation platform, with the hardware environment configured as an Intel(R) Xeon(R) Silver 4210R CPU, 64 GB of RAM (Lenovo Beijing, China), and a Windows Server 2016 Standard operating system. In the experimental design, a heterogeneous deployment scenario is constructed to compare and analyze the VF-GPTHOA with DE [44], MA [45], GWO [46], SSA [47], and the HOA in terms of key performance indicators (CR, CE, OR, and AMD) for sensor network coverage optimization. The relevant algorithm parameters are set in accordance with the original papers. Among them, DE is a classical global optimization algorithm in the field of evolutionary computation which has a mature parameter adjustment mechanism. MA is a new swarm intelligence algorithm proposed in recent years, which simulates mayfly mating and flight behavior and performs well in continuous optimization problems. GWO and SSA are designed based on the social hierarchy of animals and the foraging behavior of birds, respectively, and are representative methods in the field of swarm intelligence. These four comparison algorithms include classical and cutting-edge methods and are widely used to solve complex optimization problems, which can comprehensively verify the performance advantages of the new method.

The simulation experiment sets up deployment schemes for sensor nodes of varying scales to further investigate the algorithm’s adaptability to different scenarios. To reduce the impact of random errors on the results, it performs 20 independent repeated samples for each experiment by using the Monte Carlo method. The detailed experimental parameter configurations are shown in Table 4.

### 6.1. Experiment 1

**Figure 11:**
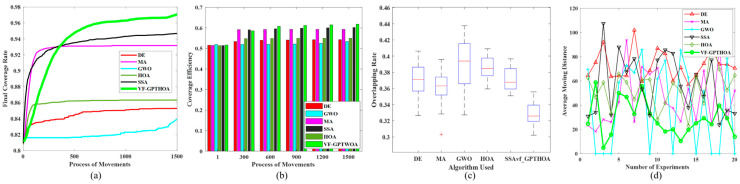
Comparison of performance metrics of six algorithms. (**a**) The evolution of the CR; (**b**) The evolution of the CE; (**c**) The statistical results of the OR; (**d**) The statistical results of the AMD.

In this experiment, 20 heterogeneous sensor nodes with sensing radii of 10 m, 8 m, and 6 m are deployed in a monitoring area of 40 m × 200 m. According to the optimal deployment principle, these 60 sensor nodes can theoretically achieve full coverage of the monitoring area. The experimental results show that the VF-GPTHOA achieves over 96% CR. After optimizing heterogeneous sensor node deployment, the CR, CE, OR, and AMD performance metrics obtained from the VF-GPTHOA are evaluated and compared with the other five algorithms. The performance differences among the algorithms are shown in Figure 11, based on the mean statistics of 20 independent experiments.

Figure 11a,b shows the evolution of the CR and CE for the six algorithms with the number of iterations. Figure 11c,d present the statistical results of the OR and AMD after 20 iterations of optimization by each algorithm during the experiment. Figure 11a shows that as the number of iterations increases, the CR of all six algorithms increases. Among them, the convergence curves of MA and SSA rise sharply in the early stages but clearly get trapped in local optima in the later stages. In contrast, the convergence curve of the VF-GPTHOA shows a steady increase, indicating that the algorithm combines fast convergence with the ability to escape local optima. This is demonstrated by its ability to avoid local optima even in the later stages of the algorithm. The superior performance of the VF-GPTHOA can be primarily attributed to its virtual force mechanism, which guides sensor nodes in directional migration through the attractive force exerted by uncovered grid points. In contrast, due to its single position update mechanism, the original HOA is prone to getting trapped in local optima and fails to achieve a global optimum. Experimental data show that the final iteration CR of the VF-GPTHOA reaches 97.07%, representing improvements of 13.81%, 4.18%, 15.81%, 2.52%, and 12.44% over DE, MA, GWO, SSA, and the HOA, respectively. In terms of the CE metric, the VF-GPTHOA leads with a value of 0.62, showing improvements ranging from 2.52% to 15.73% compared with the other algorithms. In terms of the OR metric, after 20 experimental repetitions, the OR value of the VF-GPTHOA remains stable within the range of 27–35%, fully verifying its optimization stability. For the AMD index, the VF-GPTHOA optimizes nodes’ initial and terminal position allocation before and after coverage optimization by the GPTHOA, significantly reducing the node migration distance.

**Figure 12:**
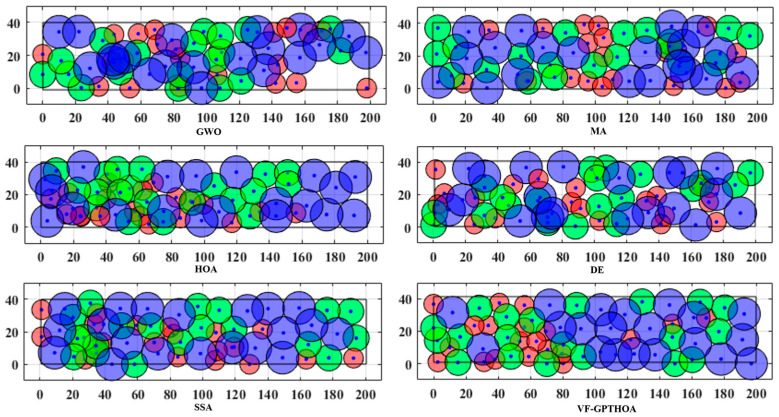
Visualization of coverage effect of six algorithms.

To visually demonstrate the performance of the VF-GPTHOA, Figure 12 shows the deployment results after 1500 iterations for six algorithms. It is clearly evident that VF-GPTHOA optimization achieves the best coverage results. Table 5 and Table 6 present the average results and standard deviation of the CR, CE, OR, and AMD after 20 experimental tests for the six algorithms, respectively. The VF-GPTHOA performs the best in all four evaluation metrics.

### 6.2. Experiment 2

**Figure 13:**
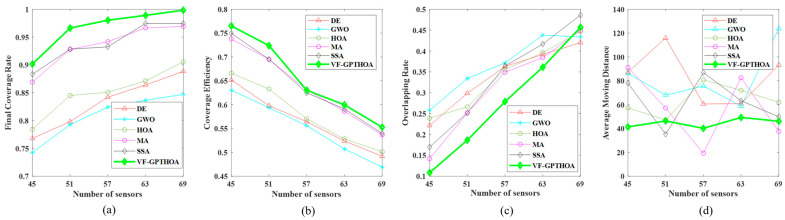
Relationship between performance metrics and number of sensor nodes. (**a**) Comparison of the CR; (**b**) Comparison of the CE; (**c**) Comparison of the OR; (**d**) Comparison of the AMD.

To address factors such as the actual physical characteristics of sensor node deployment locations and the uncertainty in the number of nodes, a multi-node deployment scheme is implemented within the target monitoring area to validate the proposed algorithm’s optimization performance and generalization ability. The experiment is conducted in a 40 m × 200 m monitoring area, with 45, 51, 57, 63, and 69 nodes being deployed. The VF-GPTHOA is compared with five other algorithms—DE, MA, GWO, SSA, and the HOA—across four performance metrics: CR, CE, OR, and AMD. The experimental results are shown in Figure 13.

After optimization with the six algorithms, the variation patterns of each metric with the number of sensor nodes show significant differences. As shown in Figure 13a, with the increase in the number of nodes, the CR values of all algorithms show an upward trend. The VF-GPTHOA consistently outperforms DE, MA, GWO, SSA, and the HOA for the same number of nodes in terms of the CR. As shown in Figure 13b, for the CE metric, all six algorithms exhibit a gradual decrease as the number of nodes increases, indicating a reduction in the uniformity of network node distribution and an increase in node redundancy. Notably, for the same node scale, the VF-GPTHOA, with its higher CR values, demonstrates superior CE performance compared with the other five algorithms, indicating lower coverage redundancy and better node distribution uniformity.

In terms of the OR metric, as shown in Figure 13c, with the network coverage approaching saturation, the OR values of all six algorithms increase with the number of nodes. When the number of deployed nodes is small, higher node dispersion helps improve network coverage. However, when the number of nodes exceeds 63, the network approaches full coverage, and further increases in nodes lead to intensified overlap in the sensing areas. As a result, the OR value of the VF-GPTHOA gradually becomes higher than that of DE, MA, and GWO. Figure 13d shows that the AMD metric of the VF-GPTHOA fluctuates less with the number of nodes, reflecting the stability advantage of the VF-GPTHOA. Regardless of the node deployment scale, the AMD of the VF-GPTHOA is always lower than that of the comparison algorithms. This is because other algorithms prioritize optimizing the CR and neglect the AMD metric, causing their AMD values to remain relatively high.

## 7. Conclusions

This paper investigates the problem of deployable and coverage enhancement of mobile heterogeneous wireless sensor nodes in bridge monitoring scenarios. It proposes a sensor node coverage optimization algorithm based on virtual force and the improved HOA called the VF-GPTHOA. Building upon the original HOA, the algorithm’s stability and global exploration capability are improved by introducing the theory of optimal point sets and the t-distribution perturbation of heterogeneous degrees of freedom. At the same time, by deeply analyzing the virtual force interaction mechanism between sensor nodes, the virtual force acting on the nodes is used as a perturbation factor for updating the hiker’s position. The virtual force and the step size decrease gradually with the number of iterations, further improving the coverage performance and avoiding oscillations in the later stages of the algorithm. After optimizing the node positions, the GPTHOA finds the shortest movement path for the nodes, reducing energy consumption. Simulation results show that the VF-GPTHOA effectively improves the coverage rate of heterogeneous sensor nodes, resulting in a more uniform node distribution than the other five algorithms.

It should be noted that this study still has the following limitations: First, the Boolean model uses binary coverage decision (complete coverage or no coverage) and ignores signal attenuation and obstacle interference, which leads to the overestimation of the effective coverage area and cannot reflect the actual change in the perceptual gradient in the complex bridge structure environment. Future research will extend this approach by using a probabilistic sensing model to enhance practical applicability. Second, this paper only presents simulation results. In the future, further validation of the proposed method’s practical applicability is required on a physical bridge or test platform. Third, the VF-GPTHOA faces scalability or time complexity issues, which need to be addressed in the future. Fourth, the deployment of Wireless Sensor Networks is essentially a multimodal multi-objective optimization problem. How to achieve Pareto-optimal deployment in the monitoring area through multi-objective algorithms will be a direction worthy of further exploration in the future.

## Figures and Tables

**Table 1:** CEC2022.

	No.	Functions	$F_{i}^{*}$
Unimodal Function	1	Shifted and full Rotated Zakharov Function	300
Basic Functions	2	Shifted and full Rotated Rosenbrock’s Function	400
Basic Functions	3	$\mathrm{Shifted}\;\mathrm{and}\;\mathrm{full}\;\mathrm{Rotated}\;\mathrm{Expanded}\;\mathrm{Schaffer}\text{’}s\;f_{6}$ Function	600
Basic Functions	4	Shifted and full Rotated Non-Continuous Rastrigin’s Function	800
Basic Functions	5	Shifted and full Rotated Levy Function	900
Hybrid Functions	6	Hybrid Function 1 (N = 3)	1800
Hybrid Functions	7	Hybrid Function 2 (N = 6)	2000
Hybrid Functions	8	Hybrid Function 3 (N = 5)	2200
Composition Functions	9	Composition Function 1 (N = 5)	2300
Composition Functions	10	Composition Function 2 (N = 4)	2400
Composition Functions	11	Composition Function 3 (N = 5)	2600
Composition Functions	12	Composition Function 4 (N = 6)	2700
$\mathrm{Search}\;\mathrm{range}:\;{\left[-100,100\right]}^{D}$			

**Table 2:** The mean, maximum, minimum, and Friedman mean of the optimal solutions obtained by the four algorithms in different dimensions (the best values for each evaluation criterion are in bold).

Function	Dim	SSOA			LCA			HOA			GPTHOA		
		Best	Mean	Worse	Best	Mean	Worse	Best	Mean	Worse	Best	Mean	Worse
F1	10	6.29E+03	1.07E+04	1.74E+04	8.59E+03	1.04E+04	1.08E+04	1.34E+03	3.75E+03	7.71E+03	**1.01E+03**	**2.32E+03**	**4.29E+03**
	20	2.63E+04	6.11E+04	2.53E+05	5.17E+04	9.06E+04	1.34E+05	1.08E+04	1.96E+04	3.06E+04	**1.02E+04**	**1.67E+04**	**2.33E+04**
F2	10	7.24E+02	1.19E+03	1.83E+03	7.83E+02	2.01E+03	4.62E+03	4.08E+02	5.27E+02	7.17E+02	**4.05E+02**	**4.48E+02**	**5.09E+02**
	20	1.34E+03	2.04E+03	3.03E+03	2.12E+03	3.71E+03	5.90E+03	6.52E+02	9.42E+02	1.22E+03	**5.30E+02**	**6.83E+02**	**8.77E+02**
F3	10	6.38E+02	6.53E+02	6.73E+02	6.43E+02	6.68E+02	6.87E+02	6.10E+02	6.21E+02	6.36E+02	**6.02E+02**	**6.07E+02**	**6.15E+02**
	20	6.71E+02	6.86E+02	7.02E+02	6.56E+02	6.94E+02	7.14E+02	6.33E+02	6.46E+02	6.60E+02	**6.13E+02**	**6.28E+02**	**6.48E+02**
F4	10	8.40E+02	8.56E+02	8.72E+02	8.51E+02	8.72E+02	8.89E+02	8.04E+02	8.21E+02	8.40E+02	8.10E+02	8.23E+02	**8.35E+02**
	20	9.55E+02	9.80E+02	1.00E+03	9.85E+02	1.01E+03	1.04E+03	8.69E+02	8.95E+02	9.32E+02	**8.52E+02**	**8.87E+02**	**9.11E+02**
F5	10	1.15E+03	1.46E+03	1.99E+03	1.36E+03	1.93E+03	2.47E+03	9.12E+02	1.05E+03	1.28E+03	9.15E+02	**9.64E+02**	**1.03E+03**
	20	2.77E+03	3.77E+03	4.70E+03	3.42E+03	4.58E+03	5.96E+03	1.37E+03	2.16E+03	3.09E+03	**1.23E+03**	**1.93E+03**	**2.82E+03**
F6	10	3.39E+06	9.58E+07	5.87E+08	8.52E+05	1.03E+08	2.05E+08	2.89E+03	8.83E+06	1.75E+08	**1.97E+03**	**3.26E+03**	**6.60E+03**
	20	3.55E+08	1.16E+09	2.31E+09	1.98E+09	4.06E+09	8.64E+09	1.38E+06	9.66E+07	3.56E+08	**1.06E+05**	**1.45E+07**	**5.67E+07**
F7	10	2.08E+03	2.13E+03	2.17E+03	2.08E+03	2.14E+03	2.21E+03	2.02E+03	2.05E+03	2.08E+03	**2.02E+03**	**2.05E+03**	**2.08E+03**
	20	2.15E+03	2.26E+03	2.41E+03	2.21E+03	2.35E+03	2.54E+03	2.07E+03	2.13E+03	2.26E+03	**2.08E+03**	**2.12E+03**	**2.18E+03**
F8	10	2.25E+03	2.31E+03	2.54E+03	2.24E+03	2.29E+03	2.40E+03	2.21E+03	2.23E+03	2.35E+03	**2.22E+03**	**2.23E+03**	**2.23E+03**
	20	2.41E+03	2.71E+03	3.08E+03	2.32E+03	2.84E+03	4.21E+03	2.23E+03	2.30E+03	2.55E+03	**2.23E+03**	**2.24E+03**	**2.35E+03**
F9	10	2.68E+03	2.76E+03	2.82E+03	2.64E+03	2.81E+03	2.95E+03	2.59E+03	2.67E+03	2.71E+03	**2.56E+03**	**2.64E+03**	**2.69E+03**
	20	2.79E+03	3.20E+03	3.46E+03	2.75E+03	3.45E+03	4.37E+03	2.64E+03	2.78E+03	2.97E+03	**2.53E+03**	**2.67E+03**	**2.87E+03**
F10	10	2.52E+03	2.65E+03	2.77E+03	2.53E+03	2.90E+03	4.71E+03	2.50E+03	2.53E+03	2.64E+03	2.50E+03	2.55E+03	**2.64E+03**
	20	2.90E+03	6.58E+03	8.16E+03	3.35E+03	7.31E+03	8.27E+03	2.52E+03	3.69E+03	5.47E+03	**2.50E+03**	**2.86E+03**	**5.33E+03**
F11	10	3.04E+03	3.68E+03	4.48E+03	3.71E+03	4.83E+03	5.15E+03	2.76E+03	3.13E+03	3.60E+03	**2.71E+03**	**2.77E+03**	**3.16E+03**
	20	6.85E+03	8.44E+03	9.10E+03	8.91E+03	1.01E+04	1.06E+04	4.09E+03	5.78E+03	7.92E+03	**4.00E+03**	**5.24E+03**	**6.48E+03**
F12	10	2.93E+03	3.02E+03	3.13E+03	2.92E+03	3.08E+03	3.29E+03	2.89E+03	2.95E+03	3.04E+03	**2.86E+03**	**2.87E+03**	**2.89E+03**
	20	3.55E+03	3.90E+03	4.46E+03	3.69E+03	4.07E+03	4.53E+03	3.24E+03	3.44E+03	3.62E+03	**2.97E+03**	**3.03E+03**	**3.15E+03**
Friedman	10	3.1667			3.8333			1.8333			**1.1667**		
	20	3.0833			3.9166			2.0000			**1.0000**		

**Table 3:** Correspondence between deployment optimization and VF-GPTHOA.

VF-GPTHOA	Deployment Optimization
Hiker	A set of deployment schemes
Leader	Optimal deployment scheme
Population size	Number of deployment scheme
Hiker’s position	HMWS’ position
Best fitness	Maximum CR

**Table 4:** Details of simulation parameters.

Parameter		Value
BM size		40 × 200
Population size N		100
Maximum number of iterations iter_max		1500
Experiment 1	Total number of sensors	60
	Sensor type and number	20 in each of the three categories
	$\mathrm{Sensing}\;\mathrm{radii}\;R_{1},R_{2},R_{3}$	10, 8, and 6
Experiment 2	Total number of sensors	45, 51, 57, 63, and 69
	Sensor type and number	15, 17, 19, 21, 23 in each of the three categories
	$\mathrm{Sensing}\;\mathrm{radii}\;R_{1},R_{2},R_{3}$	10, 8, and 6

**Table 5:** Average results of six algorithms in 20 tests.

Indicator	DE	MA	GWO	SSA	HOA	VF-GPTHOA
CR	85.29%	93.17%	83.86%	94.68%	86.33%	97.07%
CE	0.54	0.59	0.53	0.60	0.55	0.62
OR	37.09%	36.32%	39.38%	36.77%	38.48%	32.57%
AMD/m	81.3	52.1	69.8	70.4	55.6	28.9

**Table 6:** Standard deviation of six algorithms in 20 tests.

Indicator	DE	MA	GWO	SSA	HOA	VF-GPTHOA
CR	0.0076	0.0064	0.0301	0.0091	0.0165	0.0052
CE	0.137	0.124	0.126	0.008	0.012	0.006
OR	0.0193	0.0210	0.0323	0.0141	0.0154	0.0115
AMD/m	18.92	23.12	32.62	24.61	25.93	16.36

## Data Availability

Data are contained within the article.
